# Activated STAT3 Is a Novel Regulator of the *XRCC1* Promoter and Selectively Increases XRCC1 Protein Levels in Triple Negative Breast Cancer

**DOI:** 10.3390/ijms22115475

**Published:** 2021-05-22

**Authors:** Griffin Wright, Manoj Sonavane, Natalie R. Gassman

**Affiliations:** 1 Department of Physiology and Cell Biology, University of South Alabama College of Medicine, 307 N University Blvd, Mobile, AL 36688, USA; gmw1821@jagmail.southalabama.edu (G.W.); msonavane@southalabama.edu (M.S.); 2 Mitchell Cancer Institute, University of South Alabama, 1660 Springhill Avenue, Mobile, AL 36604-1405, USA

**Keywords:** base excision repair, STAT transcription factor, STAT3, breast cancer, DNA repair, chemoresistance, cytokine, stress, XRCC1

## Abstract

Base Excision Repair (BER) addresses base lesions and abasic sites induced by exogenous and endogenous stressors. X-ray cross complementing group 1 (XRCC1) functions as a scaffold protein in BER and single-strand break repair (SSBR), facilitating and coordinating repair through its interaction with a host of critical repair proteins. Alterations of XRCC1 protein and gene expression levels are observed in many cancers, including colorectal, ovarian, and breast cancer. While increases in the expression level of XRCC1 are reported, the transcription factors responsible for this up-regulation are not known. In this study, we identify the signal transducer and activator of transcription 3 (STAT3) as a novel regulator of *XRCC1* through chromatin immunoprecipitation. Activation of STAT3 through phosphorylation at Y705 by cytokine (IL-6) signaling increases the expression of XRCC1 and the occupancy of STAT3 within the *XRCC1* promoter. In triple negative breast cancer, the constitutive activation of STAT3 upregulates XRCC1 gene and protein expression levels. Increased expression of XRCC1 is associated with aggressiveness and resistance to DNA damaging chemotherapeutics. Thus, we propose that activated STAT3 regulates XRCC1 under stress and growth conditions, but constitutive activation in cancers results in dysregulation of XRCC1 and subsequently BER and SSBR.

## 1. Introduction

Base excision repair (BER) is an essential DNA repair pathway responsible for detecting and repairing abasic sites and base lesions. X-ray cross complementing group 1 (XRCC1) is a scaffold protein in the BER pathway. Although it lacks enzymatic activity, XRCC1 has a critical role in BER through recruiting and coordinating other DNA repair proteins, like DNA polymerase β (POL β) and DNA ligase III (LIG3), at DNA damage sites. XRCC1’s facilitation of protein–protein interactions provides for overlap in functions between the BER and single-strand break repair (SSBR) pathways [1,2]. In addition to BER and SSBR, XRCC1 also participates in double-strand break repair (DSBR) through its interaction with PARP1 in the error-prone alternative non-homologous end-joining (a-NHEJ) [3,4,5], as well as in nucleotide excision repair (NER) through interaction with LIG3 [6].

Given XRCC1’s critical scaffold functions, it is unsurprising that XRCC1 is ubiquitously expressed in most tissues, though low levels of XRCC1 are found in terminally differentiated muscle cells and neurons, causing impaired BER [7,8]. In tissues that should have abundant amounts of XRCC1, its loss can have profound consequences. In mice, *Xrcc1*-deficiency is lethal at day 7 [9]. Human cells that have lower levels of XRCC1 show significant sensitivity to DNA damaging agents like methyl methanesulfonate (MMS), poly(ADP-ribose) polymerase (PARP) inhibitors, and other DNA damage response and repair inhibitors [10,11,12,13,14]. Additionally, mutations in XRCC1 that reduce its ability to bind PARP1, POLβ, or interact with DNA have been shown to increase hypersensitivity to DNA damaging agents and increase genomic instability and chromosomal aberrations, promoting transformation [15,16,17,18,19,20,21,22]. While several single nucleotide polymorphisms, R399Q and R280H, have been correlated with cancer risk, variations in the gene and protein expression levels of XRCC1 are more commonly noted, particularly in ovarian, breast, and gastric cancers [14,23,24,25,26,27,28].

In breast cancer, low expression of XRCC1 correlated with improved response to poly(ADP-ribose) polymerase (PARP) inhibitors [14,26,27]. However, the prevalence of XRCC1 deficiency was ~15%, although XRCC1 deficiency was closer to 30% when breast tumors were BRCA1 deficient [13,14]. When we examined *XRCC1* expression more closely in breast cancer using the UALCAN TCGA portal, we noted significantly increased expression of *XRCC1* in luminal (566 cases, *p* < 10^−^^12^ compared with normal) and triple negative breast cancers (TNBC, 116 cases, *p* < 0.001) [11,29]. TNBC model cell lines confirmed the overexpression of XRCC1 at the gene and protein level [11,30,31]. XRCC1 overexpression is correlated with increased aggressive features and reduced cancer-specific survival in ovarian tumor samples [23]. Overexpression of XRCC1 also promotes chemoresistance in ovarian, gastric, and gallbladder cancer cell lines [23,24,25].

Despite reports demonstrating the overexpression of XRCC1, the transcriptional regulation of XRCC1 remains poorly understood, with only two transcription regulators identified thus far. The cell cycle regulator E2F transcription factor 1 (E2F1) was the first transcription factor regulating *XRCC1* to be identified [32]. E2F1 was shown to induce a robust BER response through increased XRCC1 gene and protein expression following DNA damage induced by the alkylating agent MMS [32]. More recently, the basal transcription factor, Sp1, was confirmed to control the transcription of *XRCC1* [33]. This transcriptional regulation was shown to be governed by the DNA damage sensor ataxia telangiectasia mutated kinase (ATM). Following persistent DNA damage, ATM becomes phosphorylated and, in turn, phosphorylates Sp1. Phosphorylation of Sp1-reduced *XRCC1* expression, promoting apoptosis [33].

Expression of BER factors, like 3-alkyladenine DNA glycosylase (AAG) or N-methylpurine DNA glycosylase (MPG), 8-oxoguanine DNA glycosylase 1 (OGG1), apurinic/apyrimidinic endonuclease 1 (APE1), poly(ADP-ribose) polymerase 1 (PARP1), POL β, and now XRCC1, have been shown to influence tumor characteristics and dictate chemotherapy response [14,23,24,25,28,34,35,36,37]. While several transcription regulators have been identified for PARP1, POL β, and several DNA glycosylases, our understanding of the transcriptional regulation of these genes is still developing [38,39]. More importantly, the transcriptional regulation of *XRCC1* is poorly understood in general, and even less is known about the drivers of dysregulation in cancer.

In this study, we have identified the signal transducer and activator of transcription 3 (STAT3) as a novel transcriptional regulator of *XRCC1*. The STAT family of transcription factors play a unique role in signal transduction and are critical to mediating cellular responses to external stimuli, particularly from cytokines and mitogens. During development and growth, STAT family signaling is critical to tissue differentiation and function [40]. However, aberrant STAT3 signaling has been linked to many of the hallmarks of cancer, including cell growth, proliferation, survival, immune evasion, metastasis, and angiogenesis [41]. Here, we found that STAT3 binding at the *XRCC1* promoter is significantly higher in TNBC models but is also observed in nontumorigenic human embryonic kidney cells (HEK293T). Occupancy at the binding site is increased when STAT3 is over-expressed or activated by cytokine IL-6 signaling. Importantly, in TNBC, the constitutive activation of STAT3 drives the increased expression of XRCC1, and inhibition of STAT3 or decreased expression of STAT3 reduces the expression of XRCC1. These data indicate that STAT3 conditionally regulates *XRCC1* expression and contributes to the overexpression of XRCC1 observed in TNBC.

## 2. Results

### 2.1. XRCC1 Promoter Has Active Regions Driving Reporter Expression Not Associated with Known Transcription Factors

**Figure 1:**
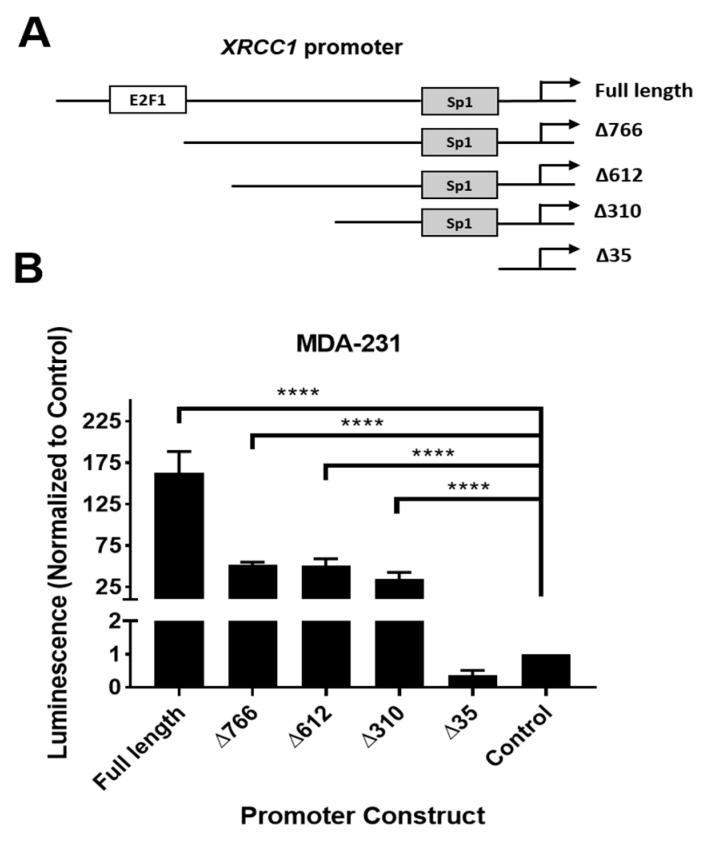
XRCC1 promoter expression in MDA-231 cells has actively transcribed regions between −612 and −35. (**A**) XRCC1 promoter fragments with known transcription factor sites were inserted into the pGL3 luciferase reporter. (**B**) Reporter plasmids were transfected into MDA-231, and luminescence was read after 24 h. **** *p* < 0.0001.

We performed a promoter-luciferase assay to identify the active regions of the *XRCC1* promoter that are capable of driving reporter expression. A series of truncated promoter regions were inserted into a pGL3-Basic vector backbone plasmid, and the luminescence values were measured in MDA-231 and HEK293T cells (Figure 1 and Supplementary Materials Figure S1). Using this assay, we identified a region capable of driving reporter expression between −612 and −35 that was not previously associated with known transcription factors [32,33].

**Figure 2:**
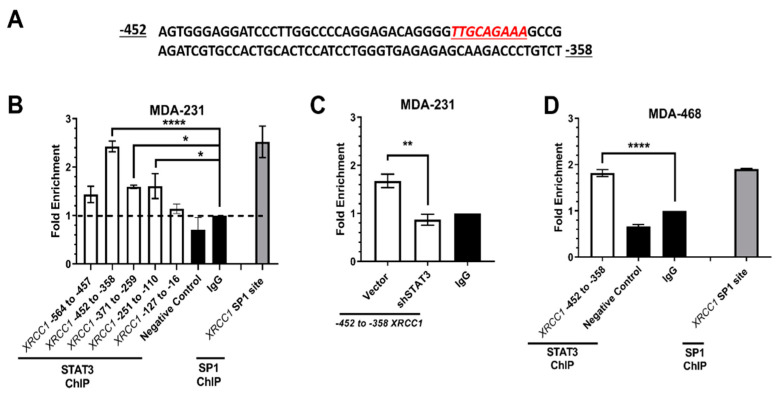
The *XRCC1* promoter contains a STAT3 binding site. (**A**) CiiiDER identified an STAT3 binding sequence within the *XRCC1* promoter (red font). (**B**) ChIP analysis of XRCC1 promoter in MDA-231 cells show a significant enrichment of STAT3 between -452 and -358. (**C**) The knockdown of STAT3 with shRNA #1 eliminates STAT3 binding within the -452 to -358 fragment. CHiP using primers specific for the SP1 binding site within the *XRCC1* promoter was performed as a positive control. (**D**) STAT3 binding also occurs within the -452 to -358 fragment of the *XRCC1* promoter in MDA-468 cells. * *p* < 0.05 ** *p* < 0.01, **** *p* < 0.0001.

To identify potential transcription factor candidates associated with this region, we performed a promoter binding ELISA (Material and Methods). Multiple potential binders within the −612 and −35 *XRCC1* promoter region were identified, including CBF, NF1, HNF4, and STAT3 (Supplementary Materials Figure S2). Of the transcription factors showing positive binding, we found STAT3 to be of particular interest due to its prominent role in multiple cancers, including triple negative breast cancer [42,43]. Additionally, we performed an in silico search using CiiiDER (www.ciiider.org, accessed on 9 November 2020) to confirm potential transcription factors. CiiiDER predicted potential transcription factor binding sites in regions of interest and confirmed a potential STAT3 binding site within the −612 to −35 region of the *XRCC1* promoter (Figure 2A) [44].

### 2.2. ChIP Confirmed the STAT3 Binding Site within the XRCC1 Promoter

Chromatin Immunoprecipitation (ChIP) was utilized to map the regions of the *XRCC1* promoter in MDA-231 that contain the STAT3 binding site. A significant fold enrichment above IgG isotype control (2.426 ± 0.11, *p* < 0.001) indicated that binding of STAT3 occurred within a 96-base pair (bp) region between -452 to -358 of the *XRCC1* promoter (Figure 2B). Knockdown of STAT3 with shRNA #1 (sh#1) in MDA-231 eliminated STAT3 binding in the -452 to -358 region (Figure 2C). STAT3 binding was further confirmed in another TNBC cell line, MDA-468, where 1.826 ± 0.066 (*p* < 0.0001) enrichment in binding at the -452 to -358 region of the *XRCC1* promoter was seen above IgG isotype control and consistent with the known SP1 binding site (Figure 2D). These findings indicate that the *XRCC1* promoter contains a STAT3 binding site with significant occupancy in TNBC cell lines.

### 2.3. STAT3 Expression Attenuated XRCC1 Expression

**Figure 3:**
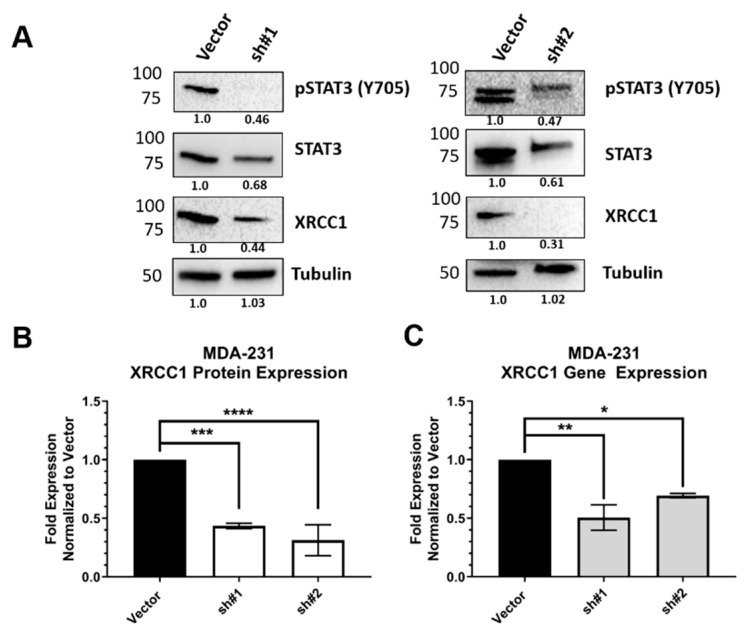
shRNA knockdown of STAT3 reduces the expression of XRCC1 in MDA-231 cells. (**A**) Representative immunoblots of phospho-STAT3 (Y705), STAT3 and XRCC1 protein expression following shRNA mediated knockdown of STAT3. α-tubulin is used as a loading control. (**B**) Quantification of protein expression changes in XRCC1 resulting from shRNA-mediated knockdown of STAT3. (**C**) Quantification of *XRCC1* mRNA expression following shRNA-mediated knockdown of STAT3. * *p* < 0.05, ** *p* < 0.01, *** *p* < 0.001, **** *p* < 0.0001.

**Figure 4:**
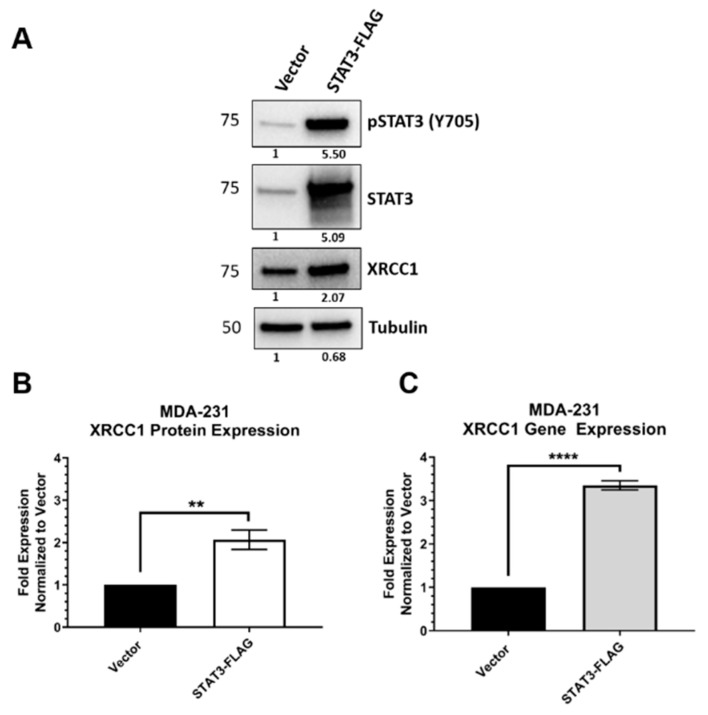
Ectopic overexpression of STAT3 increases the expression of XRCC1 in MDA-231 cells. (**A**) Representative immunoblot of phospho-STAT3 (Y705), STAT3 and XRCC1 protein expression following ectopic expression of STAT3-FLAG. α-tubulin is used as a loading control. (**B**) Quantification of protein expression changes in XRCC1 resulting from ectopic expression of STAT3-FLAG. (**C**) Quantification of *XRCC1* mRNA expression following ectopic expression of STAT3-FLAG. ** *p* < 0.01, **** *p* < 0.0001.

The transcription factor activity of STAT3 is activated by its phosphorylation at Y705 (pSTAT3) and subsequent dimerization and translocation into the nucleus. To confirm the regulatory role of STAT3 in XRCC1 expression, we first used two shRNA constructs to target STAT3 mRNA and reduce its gene expression in MDA-231. Each shRNA construct reduced the protein expression of STAT3 and reduced the presence of activated phosphorylated STAT3 (pSTAT3, Figure 3A). The reduction in STAT3 significantly reduced XRCC1 protein expression (sh#1 0.435 ± 0.019 and sh#2 0.313 ± 0.063) levels at 48 h (Figure 3B) and reduced *XRCC1* mRNA levels (sh#1: 0.506 ± 0.089 and sh#2: 0.693 ± 0.015) (Figure 3C). We then increased the expression of STAT3 by ectopic expression of a FLAG-tagged protein (Figure 4). Overexpression of STAT3 also increased the levels of activated STAT3 (pSTAT3). Increased levels of STAT3 and pSTAT3 resulted in increased XRCC1 protein (2.07 ± 0.19, Figure 4B) and mRNA (3.52 ± 0.086) levels (Figure 4C).

**Figure 5:**
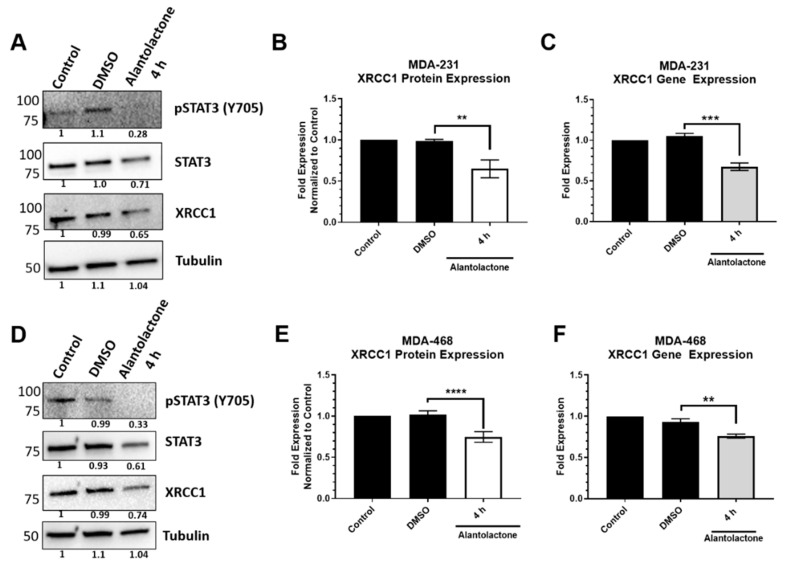
Chemical inhibition of the phosphorylation of STAT3 at Y705 by alantolactone decreases the expression of XRCC1 in MDA-231 and MDA-468 cells. (**A**) Representative immunoblots of phospho-STAT3 (Y705), STAT3 and XRCC1 protein expression after 4 h of exposure to 15 µM alantolactone in MDA-231 cells. α-tubulin is used as a loading control. (**B**) Quantification of protein expression changes in XRCC1 resulting from 4 h of alantolactone exposure in MDA-231 cells. (**C**) Quantification of *XRCC1* mRNA expression following 4 h of alantolactone exposure in MDA-231 cells. (**D**) Representative immunoblots of phospho-STAT3 (Y705), STAT3 and XRCC1 protein expression after 4 h of exposure to 15 µM alantolactone in MDA-468 cells. (**E**) Quantification of protein expression changes in XRCC1 resulting from 4 h of alantolactone exposure in MDA-468 cells. (**F**) Quantification of *XRCC1* mRNA expression following 4 h of alantolactone exposure in MDA-468 cells. ** *p* < 0.01, *** *p* < 0.001, **** *p* < 0.0001.

Given that pSTAT3 is the active transcription factor, we also specifically targeted the activated STAT3 using the pSTAT3 inhibitor, alantolactone, to block phosphorylation of STAT3 without significantly reducing the protein levels of STAT3. Pharmacological inhibition of pSTAT3 with 15 µM alantolactone produced the same trend as the shRNA constructs (Figure 5). In MDA-231, levels of pSTAT3 were reduced at 4 h of alantolactone exposure, but the protein levels of STAT3 were only slightly reduced (Figure 5A). However, XRCC1 protein (0.649 ± 0.051, Figure 5B) and *XRCC1* mRNA expression levels (0.675 ± 0.038, Figure 5C) were significantly reduced in the presence of alantolactone. A similar trend was observed in MDA-468 cells, which showed a high STAT3 protein expression level (Figure 5D). Treatment with alantolactone significantly reduced the presence of pSTAT3 (Figure 5D) and both XRCC1 protein (0.760 ± 0.026, Figure 5E) and mRNA expression levels (0.758 ± 0.020, Figure 5F). A significant reduction of XRCC1 protein and mRNA levels, along with STAT3 and pSTAT3 expression, was maintained even after 24 h of exposure to 15 µM alantolactone in the MDA-231 cells (Supplementary Materials Figure S3).

### 2.4. STAT3 Regulation Is Prevalent in TNBC

**Figure 6:**
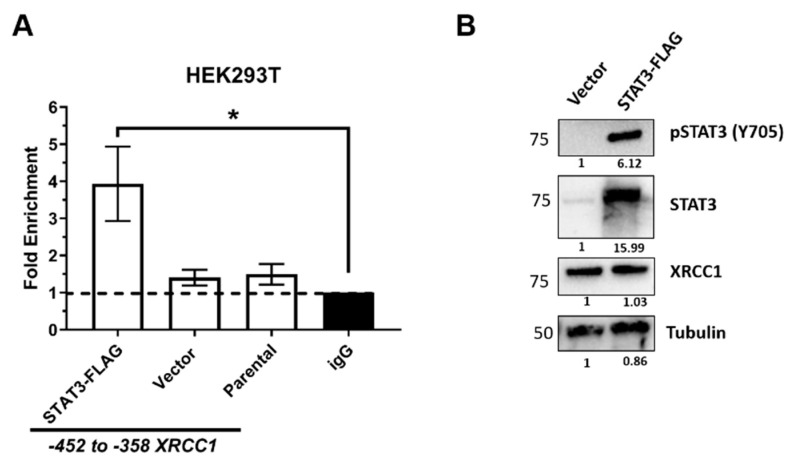
Ectopic overexpression of STAT3 increases STAT3 occupancy within the *XRCC1* promoter in HEK293T cells. (**A**) ChIP analysis of STAT3 binding to the -452 to -358 *XRCC1* promoter fragment. * *p* < 0.05 (**B**) Representative immunoblots of phospho-STAT3 (Y705), STAT3 and XRCC1 protein expression following ectopic expression of STAT3-FLAG. α-tubulin is used as a loading control.

STAT3 is constitutively activated in TNBC, but levels of pSTAT3 are more tightly regulated in normal tissues and expressed at a much lower level [43,45,46]. Therefore, to determine if STAT3 regulation of XRCC1 is specific to TNBC, we measured STAT3 binding and activation in the human embryonic kidney cell line HEK293T. The ChIP of HEK293T cells showed a very low binding occupancy of STAT3 in the -452 to -358 region (1.50 ± 0.13) (Figure 6A). Immunoblotting also showed low levels of STAT3 and pSTAT3 in these cells (Figure 6B and Supplementary Materials Figure S4). Ectopic expression of the FLAG-tagged STAT3 increased STAT3 and pSTAT3 and subsequently increased the binding occupancy of STAT3 in the -452 to -358 region (3.93 ± 0.82, Figure 6A). Interestingly, the ectopic expression of STAT3 in HEK293T did not significantly increase XRCC1 protein content (1.03 ± 0.035), indicating that STAT3 has a less significant role in regulating XRCC1 expression in HEK293T and occurs through a cancer-related, possibly TNBC-specific, mechanism.

**Figure 7:**
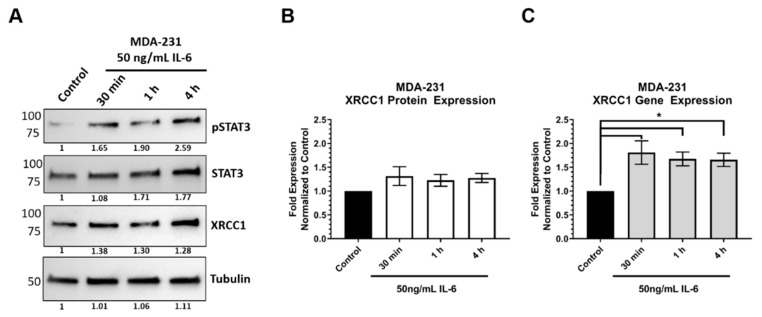
IL-6 increases phospho-STAT3 and increases the expression of XRCC1 in MDA-231. (**A**) Representative immunoblot of phospho-STAT3 (Y705), STAT3 and XRCC1 protein expression after 30 min, 1 and 4 h of exposure to 50 ng/mL IL-6. α-tubulin is used as a loading control. (**B**) Quantification of protein expression changes in XRCC1 resulting from 50 ng/mL IL-6 exposure. (**C**) Quantification of *XRCC1* mRNA expression following 50 ng/mL IL-6 exposure. * *p* < 0.05.

To understand the specificity for TNBC, we examined physiologically relevant mechanisms for STAT3 regulation of *XRCC1* in TNBC. IL-6 is a negative prognostic marker in breast cancer patients mainly due to its role in regulating STAT3 and its downstream targets promoting tumor cell proliferation, survival, and angiogenesis [43,47]. IL-6 is known to increase phosphorylation of STAT3 through JAK activation. Therefore, we examined if IL-6 activated STAT3 and resulted in changes in *XRCC1* expression. Following exposure to IL-6 (50 ng/mL) for 30 min, 1, and 4 h, a significant increase in pSTAT3 was observed in MDA-231 cells and correlated with an increase in expression of XRCC1 at both the gene and protein levels (Figure 7).

**Figure 8:**
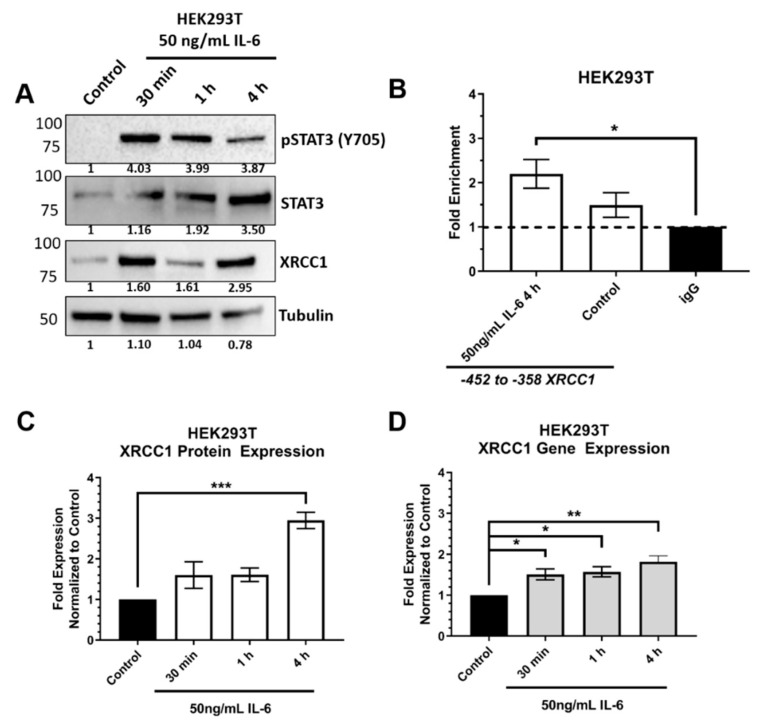
IL-6 increases phospho-STAT3, increases the occupancy of STAT3 at the *XRCC1* promoter, and increases the expression of XRCC1 in HEK293T. (**A**) Representative immunoblot of phospho-STAT3 (Y705), STAT3 and XRCC1 protein expression after 30 min, 1 and 4 h of exposure to 50 ng/mL IL-6. α-tubulin is used as a loading control. (**B**) ChIP analysis shows that IL-6 increases the STAT3 occupancy on the *XRCC1* promoter. (**C**) Quantification of protein expression changes in XRCC1 resulting from 50 ng/mL IL-6. (**D**) Quantification of *XRCC1* mRNA expression following 50 ng/mL IL-6. * *p* < 0.05, ** *p* < 0.01, *** *p* < 0.001.

We further confirmed the induced activation of pSTAT3 and regulation of *XRCC1* expression in HEK293T cells (Figure 8). HEK293T cells exposed to IL-6 showed increased activation of STAT3 (Figure 8A) and significantly increased STAT3 occupancy at the *XRCC1* promoter (2.20 ± 0.26, Figure 8B). XRCC1 gene and protein expression also increased in HEK293T cells exposed to IL-6 (Figure 8C,D), supporting the conditional regulation of XRCC1 by STAT3 after physiological stress.

We also confirmed that pSTAT3 activation by the mitogen, epidermal growth factor (EGF), would also increase the gene and protein expression of XRCC1 in MDA-231 cells (Supplementary Materials Figure S5). Exposure to 30 ng/mL EGF activated STAT3, as well as the epidermal growth factor receptor (EGFR), and increased the protein and gene expression of XRCC1 within 24 h of exposure (Supplementary Materials Figure S5).

## 3. Discussion

Dysregulation of DNA repair proteins is a hallmark of cancer. Changes in the expression of DNA repair proteins can increase susceptibility to DNA damaging therapies or increase chemoresistance [11,27,30]. While various factors can regulate gene expression, transcription factors play a critical role in basal transcription and response to stress and stimuli. Despite the critical role BER has in addressing exogenous and endogenous threats, our knowledge of the transcription factors that regulate BER factors is lacking [38,39]. Surprisingly, only two transcription factors, E2F1 and Sp1, have been identified in the transcriptional regulation of *XRCC1* [32,33].

In this study, we have identified a novel regulator of *XRCC1*, STAT3, which selectively upregulates XRCC1 in TNBC. Previously, we showed that alterations in XRCC1 gene and protein expression occur across a panel of TNBC cell lines [11]. Here, we determined that STAT3 binds within the *XRCC1* promoter in MDA-231, MDA-468, and HEK293T cells (Figure 2, Figure 6 and Figure 8). Yet, the site only has significant occupancy when STAT3 is activated. Constitutive activation of STAT3 occurs in TNBC cells and is reflected by increased occupancy at the binding site (Figure 2). However, pSTAT3 is low in the HEK293T cells, and occupancy at the STAT3 binding site in the *XRCC1* promoter is similarly low (Figure 6). This conditional regulation may explain why the promoter assay shows stable output between -612 and -310, despite the STAT3 site being deleted in the -310 construct (Figure 1 and Supplementary Materials Figure S1).

shRNA-mediated knockdown of STAT3 significantly reduced both the gene and protein expression of XRCC1 (Figure 3). Importantly, it also reduced the occupancy at the STAT3 binding site within the *XRCC1* promoter in MDA-231 cells (Figure 2C). We further confirmed the dependence on activated, phosphorylated STAT3 (Y705) by chemical inhibition with alantolactone. Alantolactone targets the SH2 domain of STAT3 and prevents phosphorylation at Y705 [48]. In the presence of 15 μM alantolactone, we again showed a significant reduction in both the gene and protein expression of XRCC1 (Figure 5 and Supplementary Materials Figure S3).

Given that TNBC cell lines showed higher levels of pSTAT3, we examined physiologically relevant stimuli that could lead to activated STAT3 and increased XRCC1 expression. Several reports have demonstrated that inflammatory signaling through IL-6R promotes constitutive activation in TNBC [47,49,50]. These signaling events are proposed to play a role in breast cancer development and progression through aberrant signaling [47]. Using IL-6, we demonstrated that STAT3 is activated in HEK293T and MDA-231 and subsequently increases the gene and protein expression of XRCC1 (Figure 7 and Figure 8). More importantly, we showed that IL-6 increased the occupancy of STAT3 at the STAT3 binding site within the HEK293T *XRCC1* promoter, which showed low occupancy under normal growth conditions (Figure 8).

These data provide evidence that STAT3 is a conditional regulator of *XRCC1* in response to stress and inflammatory signals. Under normal physiological conditions, activation of STAT3 is tightly controlled by several intrinsic inhibitors, including protein tyrosine phosphatases, the suppressors of cytokine signaling, and the protein inhibitor of activated STAT [42]. These regulatory mechanisms allow STAT3 to exert its physiological functions and limit the aberrant signaling seen in cancer. The low level of STAT3 and pSTAT3 in the HEK293T cells confirm the tightly checked role of STAT3 in these nontumorigenic cells and also that *XRCC1* expression is not driven by STAT3 in this cell line (Figure 6). Interestingly, the ectopic overexpression of STAT3 increased the levels of pSTAT3 and the occupancy of STAT3 at the *XRCC1* promoter in HEK293T but did not increase the expression of XRCC1 (1 vs. 1.03, Figure 6). However, after stimulation with IL-6, we see a dramatic increase in the presence of activated pSTAT3 and increased XRCC1 protein and gene expression (Figure 8). Therefore, the expression and activation of STAT3 alone is not enough to stimulate the transcription of *XRCC1* in this nontumorigenic cell line.

Transformation involves numerous cellular and genomic changes that reduce inhibitions on growth and proliferation signals. These changes also reduce apoptotic signaling, cell cycle control mechanisms and alter DNA damage response. As a result, we see overexpression of STAT3 being sufficient to drive *XRCC1* expression in MDA-231 cells (Figure 4), as well as stimulation by IL-6 and EGF (Figure 7 and Supplementary Materials Figure S5). These results are consistent with two recent studies, which examined the role STAT3 plays in regulating growth and invasion in TNBC cell lines [51,52]. Both studies used CHiP-seq to examine the transcriptional regulation of genes by STAT3. While their focus was on proliferation, migration and invasion genes, examination of the CHiP-seq results (GSE85579 and GSE152203) at the *XRCC1* promoter showed STAT3 binding sites within the MDA-MB-231, MDA-MB-468, and HCC70 cells [51,52]. These CHiP-seq results in basal-like TNBC cell lines support our findings of higher expression and activation of STAT3 resulting in increased XRCC1 expression [11,51,52]. Further validation of the pSTAT3 dependence of these sites is needed to better understand the impact of the conditional regulation of XRCC1 by pSTAT3 in TNBC.

The more blunted response in HEK293T cells and reports of under-expression of XRCC1 in hormone-positive breast cancers suggest that activated STAT3 regulation of XRCC1 may be highly tissue specific and dependent on exogenous signals like IL-6 or EGF [27]. This tissue specificity is supported by the finding that stimulation by IL-6 is more robust than ectopic expression alone (~3-fold vs. 2-fold) in HEK293T. The difference in expression from IL-6 vs. ectopic STAT3 may be related to the downregulation of inhibiting factors such as SOCS3 or could reflect the additional changes in redox balance and reactive species induced by IL-6 [47,53,54,55]. Additional studies are needed to differentiate these contributors in the STAT3-related transcriptional control of *XRCC1*, although, in all likelihood, these mechanisms are probably inter-related.

In TNBC, IL-6 plays a critical role in breast cancer growth and maintenance [47,55,56]. TNBC tumor cells can autonomously produce IL-6, resulting in the constitutive activation of STAT3 [55,56]. Activated STAT3 acts as a transcription factor controlling the expression of genes involved in regulating cell proliferation, anti-apoptosis, migration, invasion, angiogenesis, chemoresistance, immune escape, and autophagy [43]. Here, for the first time, we have linked STAT3 activation by cytokines and stress factors to the regulation of a DNA repair protein, XRCC1. In our previous work with TNBC cell lines, we noted the over-expression of XRCC1 in pre-clinical TNBC cell lines, which contrasted with previous reports on hormone-positive breast cancers noting a deficiency in XRCC1 expression [11,14,27,30]. By examining the effects of XRCC1 over-expression, we noted resistance of the alkylating agents to MMS in highly over-expressed XRCC1 cell lines. Other reports have associated the upregulation of XRCC1 with increased risk of breast cancer; poor survival across low and high-risk breast cancer subtypes; increased tumor aggressiveness; and resistance to cisplatin, PARP inhibitors, and ionizing radiation [9,26,27,57]. However, the mechanism driving the overexpression of XRCC1 in TNBC and other cell lines has not been identified.

We were able to reverse MMS resistance through shRNA-mediated knockdown of XRCC1 expression [11]. Additionally, the under-expression of XRCC1, seen in some hormone-positive breast cancers, is correlated with increased sensitivity to chemotherapeutics, including ionizing radiation, cisplatin, and PARP inhibitors [14,26,27,35]. Together, these results suggest that attenuation of XRCC1 expression influences breast cancer etiology and response to therapy. While we have identified pSTAT3 as a novel regulator of XRCC1 in TNBC, it is also likely that activated STAT3 regulates *XRCC1* under stress and growth conditions in nontumorigenic cells. However, it is not until pSTAT3 levels become dysregulated that sustained increases in XRCC1 expression and subsequently changes in BER and SSBR would be observed, contributing to chemoresistance and tumor aggressiveness. The constitutive activation of STAT3 in TNBC allowed this regulation to be identified more readily.

This work illuminates the complex regulatory mechanisms of BER proteins like XRCC1. Dysregulation of DNA repair proteins is a hallmark of cancer, yet basal and stress-induced regulatory mechanisms for these proteins are poorly delineated [38,39]. Here, we have identified a stress-specific regulatory mechanism for increasing the protein levels of XRCC1, which becomes dysregulated in TNBC and potentially other cancers.

## 4. Materials and Methods

### 4.1. Cell Culture

MDA-MB-231(MDA-231), MDA-MB-468 (MDA-468), and HEK293T were purchased from the American Type Culture Collection (ATCC HTB-26, HTB-132, and CRL-3216, respectively; Manassas, VA, USA) within the last 24 months and passaged < 15 times for all experiments. Cells were tested biweekly during experiments for mycoplasma contamination using the Lonza MycoAlert^®^ (Lonza #LT07-318). MDA-231 and MDA-468 cells were grown in DMEM High Glucose + GlutaMAX™ (Life Technologies, Carlsbad, CA, USA, #10566016) and supplemented with 1% sodium pyruvate (Life Technologies, #11360070) and 10% FBS (Premium Select, R&D systems, Minneapolis, MN, USA). HEK293T cells were grown in DMEM High Glucose + L-Glutamine (HyClone, Logan, UT, USA, # SH30022.01) and supplemented with 1% sodium pyruvate (Life Technologies #11360070) and 10% FBS. Cells were maintained in a humidified 37 °C incubator with 5% carbon dioxide.

### 4.2. Promoter Luciferase Assay

Transcriptional activity at the *XRCC1* promoter was measured using a dual promoter-luciferase assay similar to Chen et al. [32]. The pGL3 plasmid containing the full-length *XRCC1* promoter from Chen et al. was provided by Dr. Charles Lopez (Oregon Health Sciences University, Portland, OR, USA). XRCC1 promoter fragments XRCC1, ∆766, ∆612, ∆310, and ∆35 were cloned using *XRCC1* promoter-specific primers (Table 1) from genomic DNA harvested from MDA-231 cells. Promoter PCR fragments were digested with Nhel Anza™ (Thermo Fisher Scientific, Waltham, MA, USA, #IVGN0066) and NcoI Anza™ (Thermo Fisher Scientific#IVGN0026) and then ligated into a pGL3 plasmid backbone with Anza™ T4 DNA Ligase (Thermo Fisher Scientific #IVGN2104). The final plasmid constructs with the correct promoter fragment insertion were confirmed by Sanger sequencing by Eurofins. MDA-231 cells were transfected with 0.4 µg of plasmid DNA and 0.1 µg of pRSV β galactosidase plasmid DNA using Jetprime (Polyplus transfection, New York, NY, USA, #114−15, 1:6). HEK293T cells were transfected with 0.4 µg of plasmid DNA and 0.1 µg of pRSV β galactosidase plasmid DNA (Promega, Madison, WI, USA) using Jetprime transfection reagent (1:2). pGL3 was used as a negative control to ensure the assay was working correctly. Using the β-Galactosidase Enzyme Assay System with Reporter Lysis Buffer (Promega #E2000) and the Luciferase Assay System (Promega #E1500), transfected cells were lysed 24 h after transfection, and luminescence and absorbance were collected using an Infinite^®^ M1000 PRO, TECAN (Mannedorf, Switzerland). Luminescence values were normalized to the respective β-galactosidase absorbance to control for transfection efficiency. The assay was performed in parallel plates in technical triplicate over three biological replicates. Results represent the average of the three biological replicates ± standard error of the mean (SEM).

### 4.3. Promoter Binding ELISA

Potential transcription factors binding the *XRCC1* promoter were identified using the transcription factor binding array (Signosis Santa Clara, CA, USA #FA-1001-NE). Following the manufacturers’ instructions. Nuclear extracts were isolated from MDA-231, and the binding of transcription factors was tested using the *XRCC1* full length and *XRCC1* ∆35 PCR products described in the promoter luciferase sections, with the primers detailed in Table 1. The promoter binding ELISA was performed with two biological replicates, using Sp1 as a positive binding control.

### 4.4. Chromatin Immunoprecipitation (ChIP)

MDA-231, MDA-468, and HEK293T cells were grown to confluency in a 150 mm dish. The cells were crosslinked by the addition of 1% formaldehyde in DMEM with gentle rocking at room temperature (RT ~23 °C) for 8–10 min. Then, 0.1 M glycine was added for 5 min at RT to quench the formaldehyde. The cells were washed with cold 1× phosphate-buffered saline (PBS) and subsequently lysed with 1 mL of farnham lysis buffer (5 mM HEPES pH 8.0, 85 mM KCl. 0.5% NP-40) for 20 min on ice, then pelleted by centrifugation at 2000 rpm and resuspended in RIPA buffer (50mM Tris-HCl pH 8, 150mM NaCl, 1% sodium deoxycholate, 1mM EDTA, 0.1% SDS, 1% Triton X-100) for 20 min. Isolated chromatin was then sonicated on ice at an amplitude of 12 on a Misonix S-4000 with 15 s on/50 s off for a total process time of 2.5 min for MDA-231 and MDA-468 and amplitude of 10 on a Misonix S-4000 with 15 s on/50 s off for a total process time of 3.5 min for HEK293T. Chromatin was incubated overnight at 4 °C on a rotator using an anti-STAT3 antibody diluted to manufactures’ recommendations for chromatin immunoprecipitation (Cell Signaling Technology, Danvers, MA, USA #9131S), an anti-Sp1 antibody (Abcam Cambridge, MA, USA #ab13370) diluted 1:100 as a positive control, a mouse IgG isotype control (Cell Signaling Technology #5415S) and with Protein A/G magnetic beads (Thermo Fisher Scientific #88802). Magnetic beads were washed with cold LiCl wash buffer (100 mM Tris-HCl, 500 mM LiCl, 1% NP-40, 1% Triton X-100) and TE Buffer (10mM Tris-HCl pH 7.5, 0.1mM EDTA). Proteinase K (VWR Life Science Radnor, PA, USA # E195-5ML) was then added with ChIP Elution Buffer (1% SDS, 0.1 M NaHCO_3_) and incubated at 65 °C 950 rpm for 2 h. Proteinase K was then inactivated at 90 °C for 10 min. DNA was purified using a PureLink PCR Purification Kit (Life Technologies #K310002 kit). An IgV browser was used to design primers examining the occupancy across the *XRCC1* promoter (Table 2).

### 4.5. Modulated Expression of STAT3

Plasmid constructs for stable depletion of human STAT3 mRNA, pSIH-puro-STAT3 shRNA (referred two as shRNA #1), and its control were gifts from Frank Sinicrope (Addgene plasmid #26596 and #26597; Watertown, MA, USA). An additional shRNA construct specific for STAT3 (shRNA Clone ID:NM_003150.3-458s21c1 referred to as shRNA #2 hereafter) and its pLKO.1 control were purchased from Sigma-Aldrich (St. Louis, MO, USA). Both shRNA constructs and their controls were used to validate the STAT3 binding site and expression changes. MDA-231 cells were plated at 200,000 cells/well in a 6-well culture plate. After 48 h, cells were transfected with 5 μg plasmid DNA (shRNA# 1 or 2 or appropriate vector control) and FuGene 6 (Promega) at a 1:6 ratio (DNA to FuGene). Cells were allowed to recover for 48 h following transfection. STAT3 was overexpressed using a pcDNA3.1+ STAT3 ORF clone from Genscript (Piscataway, NJ, USA) that has a C-terminal Flag-tag. MDA-231 cells were plated at 200,000 cells/well in a 6-well culture plate, and HEK293T were plated in 10 cm plates at 500,000 cells/plate. After 48h, MDA-231 cells were transfected with 5 μg of plasmid DNA (STAT3-FLAG and proper vector control) and Fugene 6 (Promega) in a 1:6 ratio (DNA to Fugene). HEK239T cells were transfected with 10 μg of plasmid DNA (STAT3-FLAG and proper vector control) and Jetprime transfection agent at a 1:2 ratio (DNA to Jetprime). 48 h post-transfection, cells were rinsed with 1X PBS, plates were scraped, and the pelleted cells were stored overnight in −80 °C. Immunoblot was then performed as described below.

### 4.6. Gene Expression and qPCR

Relative gene expression was performed through mRNA isolation from MDA-231, MDA-468, and HEK293T cell lines using Invitrogen Cell to C_t_ kit (Life Technolgoies #4399002). Following the manufacturers’ recommendations, the cells were plated in a 96-well plate, and the untreated cells were grown to 75% confluency. For transfection, 0.1 μg of plasmid DNA was added with Fugene 6 transfection reagent in a 1:6 ratio (plasmid DNA to Fugene). Cells were then allowed 48h to recover before being lysed for mRNA isolation using an Invitrogen Cell to C_t_ kit (Life Technologies #4399002). The cells were then lysed, and RT-PCR was performed to produce cDNA using the reagents from the kit. After cDNA synthesis, qPCR was performed using TaqMan Gene expression primers (Table 3) and the TaqMan master mix provided with the kit (Applied Biosystems Foster City, CA, USA #4369016). The assay was performed in technical triplicate over three biological replicates. Results represent the average of the three biological replicates ± standard error of the mean (SEM).

### 4.7. Cytokine Exposure

Cytokine exposure was performed using recombinant Human IL-6 protein (R&D Systems, Minneapolis, MN, USA, #206-IL-010/CF). IL-6 was aliquoted in PBS at a concentration of 100 µg/mL and stored at −80 °C for no longer than 3 months before use, as recommended by the manufacturers. Aliquoted IL-6 was added to the cell culture medium to a final concentration of 50ng/mL. MDA-231 and HEK293T cells were plated in 15 mm dishes and cultured to 70–80% confluency. Cells were then exposed to IL-6 at a final concentration of 50ng/mL for 30 min, 1, and 4 h. Cells rinsed with 1X PBS plates were scraped, and pelleted cells were stored overnight in −80 °C. Immunoblot was performed as described below.

### 4.8. Immunoblot

Immunoblot was performed as described previously [11]. Briefly, the cells were grown in 150 mm dishes and cultured to 70–80% confluence. Cells were rinsed with PBS, scraped, stored overnight at −80 °C, then lysed. Protein content was quantified using a Bradford assay. Then, 20 µg of lysate was separated on 7.5% or 4–15% SDS Page gel (Bio-Rad #s, 4561025 and 4561084) and transferred to a nitrocellulose membrane. Membranes were blocked in 5% non-fat dry milk in Tris-buffered saline (VWR #J640-4L) containing 0.1% Tween20 (Thermo Fisher Scientific #BP337, TBS-T) and raised against the following primary antibodies: XRCC1 (1:1000 #MS434P1) from Fisher Scientific (Pittsburgh, PA, USA); STAT3 (1:1000, #9139) and pSTAT3 Y705 (1:500, #9131) from Cell Signaling Technology, Inc.; and α-tubulin (1:5000, #T9026) from Millipore Sigma (St. Louis, MO, USA) summarized in Table 4. The blots were incubated with either of the horseradish peroxidase (HRP)-labeled secondary antibodies: goat anti-rabbit-HRP or goat anti-mouse-HRP (#7074P2 and #7076S respectively) from Cell Signaling Technology, Inc. HRP antibody target proteins were detected by incubating with WesternBright Sirius (Advansta San Jose, CA, USA #K-12043-D20). All immunoblotting was conducted with three biological replicates. Where indicated, protein quantification was conducted with Image Lab Software (Bio-Rad, Hercules, CA, USA). Band intensity was normalized to loading controls and averaged over the three biological replicates with SEM presented.

### 4.9. Statistical Analysis

Assays were performed as three biological replications. One-way ANOVA and means were compared with Dunnett’s post hoc analysis. Comparison groups are indicated in graphs. All means are reported ± SEM.

## 5. Conclusions

Here, we demonstrated that activation of STAT3 through cytokine stimulation or ectopic overexpression stimulated the expression of *XRCC1.* Further, we confirmed a binding site for STAT3 within the *XRCC1* promoter with higher occupancy in triple negative breast cancer cell lines than in the nontumorigenic HEK293T. Together, these data indicate that activated STAT3 regulates XRCC1 expression, and constitutive activation of STAT3 leads to dysregulated expression of XRCC1. The consequences of this dysregulation need more investigation but likely lead to BER dysfunction, which we have previously observed in triple negative breast cancers [11].

## Figures and Tables

**Table 1:** Promoters for luciferase assay cloning.

Primer	Sequence
XRCC1 Full Length Forward	CTTACGCGTGCTAGCGGACGCAGAACCC
XRCC1 Full Length Reverse	GCGTCTTCCATGGTCACCGAGTCCTGGCTGC
XRCC1 ∆766 Forward	CTTACGCGTGCTAGCGCAAGGGGACAGAGAGAAGAG
XRCC1 ∆612 Forward	CTTACGCGTGCTAGCGAGGCCGAGGCAGGTGGATC
XRCC1 ∆310 Forward	CTTACGCGTGCTAGCGGATTTGCTTTCTCGGCTTC
XRCC1 ∆35 Forward	CTTACGCGTGCTAGCGGCCGGGGTTTGAAAGGC

**Table 2:** Chromatin Immunoprecipitation primers.

Primer	Sequence
XRCC1 -564 to -457 Forward	TGGGCAACATGGCAAGA
XRCC1 -564 to -457 Reverse	CTCCTAAGTAGCTGGGATTACAC
XRCC1 -452 to -358 Forward	AGTGGGAGGATCCCTTGG
XRCC1 -452 to -358 Reverse	ACAGGGTCTTGCTCTCTCA
XRCC1 -312 to -236 Forward	AAAGATTTGCTTTCTCGGCTTC
XRCC1 -312 to -236 Reverse	CAGTCGCGCCTCTCTTC
XRCC1 -251 to -110 Forward	TTTCTTCCAGACACCAATCCC
XRCC1 -251 to -110 Reverse	TAGCAACGAGCGTTTCCTC
XRCC1 -127 to -16 Forward	AGGAAACGCTCGTTGCTAA
XRCC1 -127 to -16 Reverse	TCGGGCCTTTCAAACCC
XRCC1 SP1 Site Forward [33]	ATTGGGAGGCGAGGCTA
XRCC1 SP1 Site Reverse [33]	TCTCCAGAGCGGGAAGAG

**Table 3:** TaqMan primers.

Gene	Primer
XRCC1	Hs00959834_m1 FAM
STAT3	Hs00374280_m1 FAM
ACTIN	Hs01060665_g1 VIC

**Table 4:** Antibodies and dilutions used for immunoblotting experiments.

Antibody	Dilution
XRCC1 (Fisher Scientific #MS434P1)	1:1000
STAT3 (Cell Signaling Technology #9139)	1:1000
Alpha-Tubulin (Sigma Aldrich #T9026)	1:5000
pSTAT3 Y705 (Cell Signaling Technology #9131)	1:500

## Data Availability

All data is contained within the manuscript.

## Supplementary Materials

The following are available online at https://www.mdpi.com/article/10.3390/ijms22115475/s1, Figure S1: *XRCC1* promoter expression in HEK293T cells has actively transcribed regions between −612 and −35; Figure S2: Promoter binding ELISA identifies STAT3 as a positive binder to the XRCC1 promoter; Figure S3: Chemical inhibition of the phosphorylation of STAT3 at Y705 by alantolactone decreases the expression of XRCC1 in MDA-231 at 4 and 24 h; Figure S4: Immunoblotting of cell lines MDA-231, MDA-468 and HEK293T; Figure S5:EGF increases phospho-STAT3 and increases the expression of XRCC1 in MDA-231.
