# Supplementary material for: Activated STAT3 Is a Novel Regulator of the XRCC1 Promoter and Selectively Increases XRCC1 Protein Levels in Triple Negative Breast Cancer
Source: Int J Mol Sci. 2021 May 22;22(11):5475. doi: 10.3390/ijms22115475 (PMC8196947; doi:10.3390/ijms22115475)
Supplement: Supplementary file 1 [file ijms-22-05475-s001.zip › ijms-1187084-supplementary.pdf]

Supplementary Information

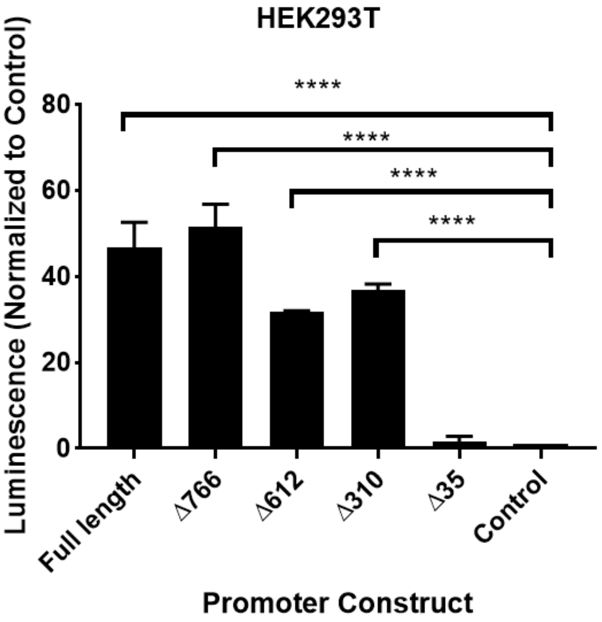

Supplementary Figure S1: ***XRCC1*** **promoter expression in HEK293T cells has actively transcribed regions between -612 and -35.** Reporter plasmids were transfected into MDA-231 and luminescence read after 24 h. ****p<0.0001

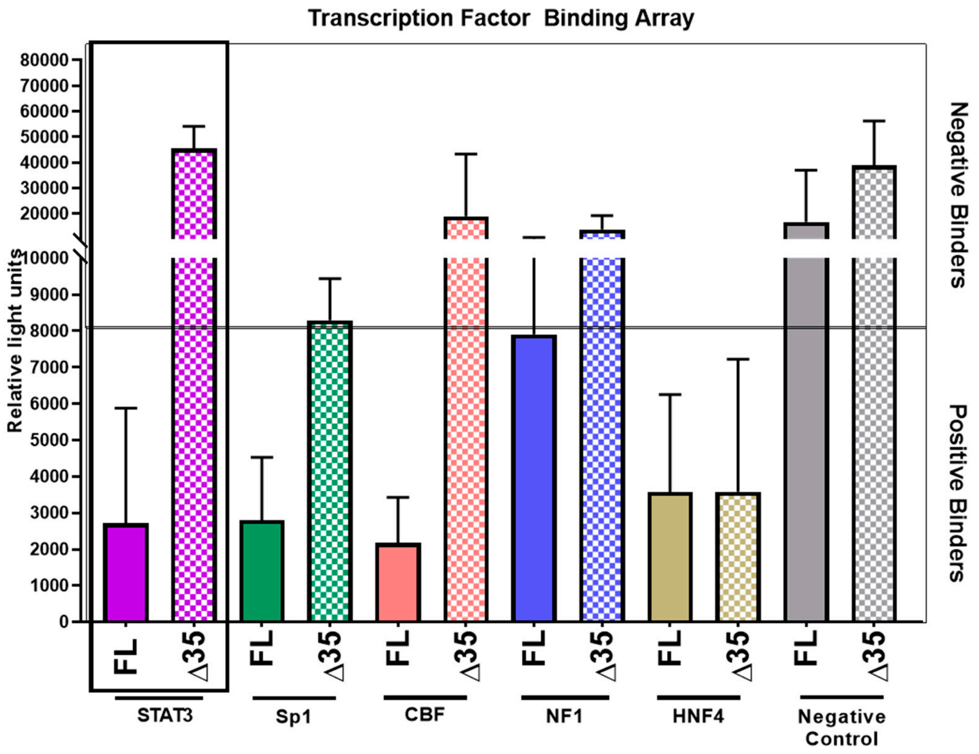

Supplementary Figure S2: Promoter binding ELISA identifies STAT3 as a positive binder to the XRCC1 promoter. STAT3 was identified as a positive binder to the XRCC1 full length promoter (FL) with no binding occurring in the Δ35 promoter construct. The graph represents 2 biological replicates.

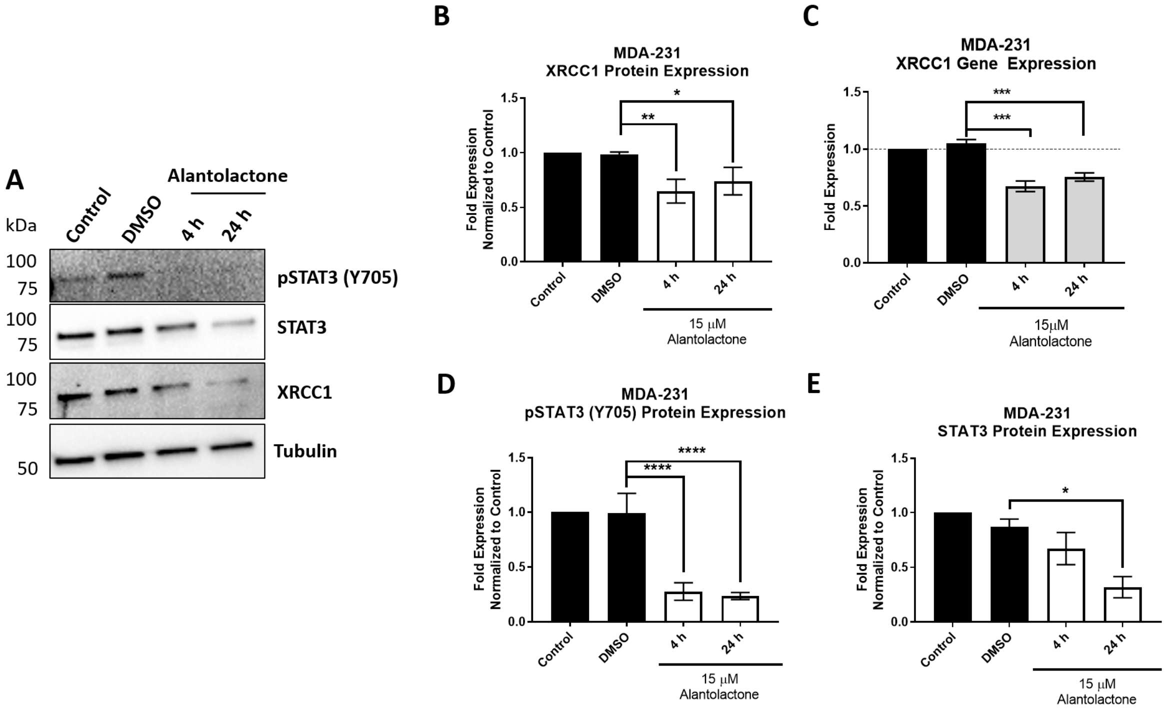

Supplementary Figure S3: Chemical inhibition of the phosphorylation of STAT3 at Y705 by alantolactone decreases the expression of XRCC1 in MDA-231 at 4 and 24 h. A) Representative immunoblots of phospho-STAT3 (Y705), STAT3 and XRCC1 protein expression after 4 and 24 h exposure to 15 μM alantolactone. α-tubulin is used as a loading control. Average quantification of three biological replicates is reported under the bands. B) Quantification of protein expression changes in XRCC1 resulting from 4 and 24 h alantolactone exposure. C) Quantification of *XRCC1* mRNA expression following 4 and 24 h alantolactone exposure. D) Quantification of protein expression changes in pSTAT3 resulting from 4 and 24 h alantolactone exposure. E) Quantification of STAT3 protein expression following 4 and 24 h alantolactone exposure.

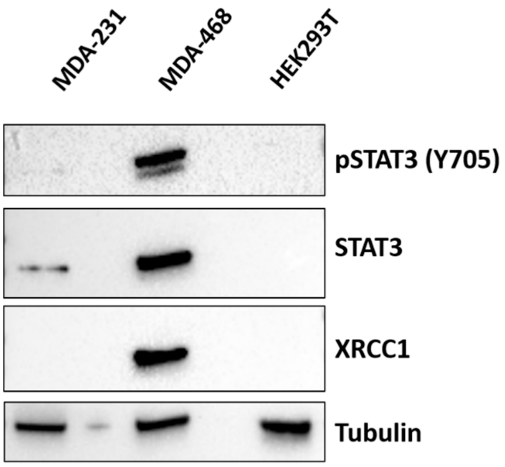

Supplementary Figure S4: Immunoblotting of cell lines MDA-231, MDA-468 and HEK293T. A) Representative immunoblot of phospho-STAT3 (Y705), STAT3, and XRCC1 protein expression. α-tubulin is used as a loading control.

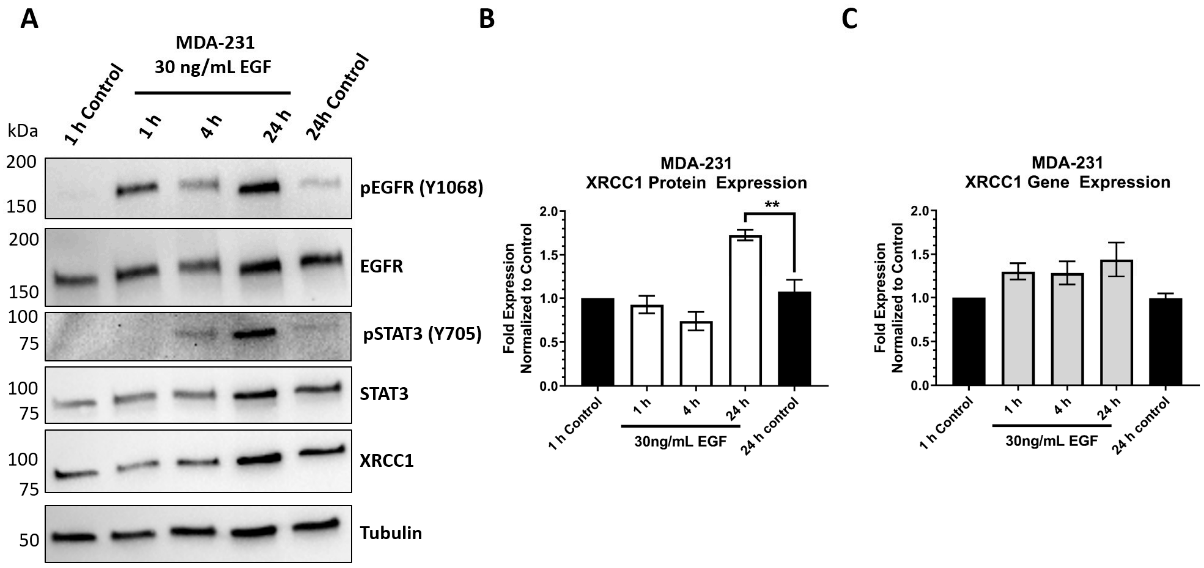

Supplementary Figure S5: EGF increases phospho-STAT3 and increases the expression of XRCC1 in MDA-231. A) Representative immunoblot of phospho-EGFR(Y1068), EGFR, phospho-STAT3 (Y705), STAT3 and XRCC1 protein expression after 1, 4, and 24 h exposure to 30 ng/mL EGF (Thermo Fisher Scientific). α-tubulin is used as a loading control. Average quantification of three biological replicates is reported under the bands. B) Quantification of protein expression changes in XRCC1 resulting from 30 ng/mL EGF. C) Quantification of *XRCC1* mRNA expression following 30 ng/mL EGF. **p< 0.01
